# Ingestion of micronutrient fortified breakfast cereal has no influence on immune function in healthy children: A randomized controlled trial

**DOI:** 10.1186/1475-2891-10-36

**Published:** 2011-04-21

**Authors:** David C Nieman, Dru A Henson, Wei Sha

**Affiliations:** 1 Human Performance Lab, North Carolina Research Campus, Kannapolis, NC 28081, USA; 2 Biology; P.O. Box 32071, Appalachian State University, Boone, North Carolina 28608, USA; 3 Bioinformatics Services Division, UNC-Charlotte, North Carolina Research Campus, Kannapolis, NC 28081, USA

## Abstract

**Background:**

This study investigated the influence of 2-months ingestion of an "immune" nutrient fortified breakfast cereal on immune function and upper respiratory tract infection (URTI) in healthy children during the winter season.

**Methods:**

Subjects included 73 children (N = 42 males, N = 31 females) ranging in age from 7 to 13 years (mean ± SD age, 9.9 ± 1.7 years), and 65 completed all phases of the study. Subjects were randomized to one of three groups--low, moderate, or high fortification--with breakfast cereals administered in double blinded fashion. The "medium" fortified cereal contained B-complex vitamins, vitamins A and C, iron, zinc, and calcium, with the addition of vitamin E and higher amounts of vitamins A and C, and zinc in the "high" group. Immune measures included delayed-typed hypersensitivity, global IgG antibody response over four weeks to pneumococcal vaccination, salivary IgA concentration, natural killer cell activity, and granulocyte phagocytosis and oxidative burst activity. Subjects under parental supervision filled in a daily log using URTI symptoms codes.

**Results:**

Subjects ingested 3337 ± 851 g cereal during the 2-month study, which represented 14% of total diet energy intake and 20-85% of selected vitamins and minerals. Despite significant increases in nutrient intake, URTI rates and pre- to- post-study changes in all immune function measures did not differ between groups.

**Conclusions:**

Data from this study indicate that ingestion of breakfast cereal fortified with a micronutrient blend for two winter months by healthy, growing children does not significantly influence biomarkers for immune function or URTI rates.

## Background

Nutrients are involved in the immune response to pathogens, facilitating cell division and the production of specific antibodies and cytokines, and in providing metabolic support for skin and mucosa physical barriers [1]. Enzymes in immune cells require the presence of micronutrients, and critical roles have been defined for nearly all nutrients, including zinc, iron, copper, selenium, and vitamins A, C, E, D, and B_6 _[2,3]. One of the earliest interactions between nutrition, immunity, and infection was established in malnourished children [4,5]. Nutritional deficiencies and immune dysfunction, with improvements measured following immunonutrition support, have been linked in several other groups including the frail elderly, patients experiencing surgery, illness, and trauma, and human immunodeficiency virus (HIV) infected individuals [6-13].

Less clear is the immune-related benefit of nutrient supplementation among healthy children and adults with no overt signs of immune system deficiencies. Among free-living adults, a wide variance in self-selected diet intake is compatible with normal immune function [14-16]. High compared to low self-selected intake of vitamins through diet or supplements by adults is not associated with altered risk of pneumonia [17,18]. Although data are limited, mixed or single micronutrient supplementation among healthy, community-dwelling adults is largely ineffective in altering innate or adaptive immune function, or in lowering respiratory infection rates [19-24].

Zinc, iron, and/or vitamin A, C, and E supplementation in young, malnourished or diseased children in developing countries reduces respiratory infection morbidity and helps counter impaired immunity [25-31]. The influence of mixed micronutrient supplementation on immune function and incidence of upper respiratory tract infections (URTI) in healthy children is largely unstudied [32]. Children suffer from a high rate of URTI, and the physiologic stress of rapid growth and suboptimal dietary quality may provide room for immune benefit through micronutrient supplementation [3]. We hypothesized that school-aged children would experience improvements in innate and adaptive immune function and a reduction in URTI during two winter months of supplementation with a mixture of immune-related micronutrients administered through a fortified breakfast cereal.

## Methods

### Subjects and research design

Seventy-three children (42 boys and 31 girls) ranging in age from 7 to 13 years, and in body mass index from 13 to 36 kg/m^2^, were recruited from local elementary schools and home school programs. Inducements included subject stipends and free results of fitness, body composition, and immune function tests. This study was conducted according to the guidelines laid down in the Declaration of Helsinki and all procedures involving human subjects/patients were approved by the university's Institutional Review Board for Human Studies. Written informed consent was obtained from all subjects and a parent. A parent for each child attended all orientation and test sessions, and assumed responsibility for home feeding, dietary recording, health logs, and transportation of their children to the laboratory.

Triceps and subscapular skinfolds were measured in each child and summed using the procedures of Lohman et al. [33]. The skinfolds were measured by one trained technician using a Lange skinfold caliper (Cambridge Scientific Industries, Cambridge, MA).

Subjects were tested for immune function pre-study, and then again two months later following a regimen of micronutrient supplementation through ingestion of a fortified breakfast cereal. The subjects used a daily health log to record symptoms of sickness using number codes. A pneumococcal vaccine was administered halfway through the study, with the antibody response measured one month later (post-study).

### Supplementation regimen

Subjects were randomized to one of three groups--low, medium, or high fortification--with breakfast cereals administered in double blinded fashion. Extruded, expanded, puffed corn cereal products with selected micronutrient blends were supplied by General Mills in coded boxes (Minneapolis, MN). For all groups, each 100 g cereal provided 400 kilocalories, 6.7 g protein, 86.7 g carbohydrate, 1.7 g fat, 3.3 g dietary fiber, 133 mg potassium, and 900 mg sodium. The "low" fortification group received the following nutrients per 100 g cereal: 0.8 mg vitamin C, 133 mg calcium, and 0.6 mg iron. The calcium provided in the low fortification cereal ensured that subjects received partial support for growing bones. The "medium" fortification group received the following nutrients per 100 g cereal: 1.3 mg thiamin, 1.4 mg riboflavin, 16.7 mg niacin, 1.7 mg vitamin B6, 5.0 μg vitamin B12, 20.0 mg vitamin C, 1,667 IU vitamin A, 667 μg folate, 500 mg calcium, 27.0 mg iron, and 12.5 mg zinc. The "high" fortification group received the same amount of nutrients as the "medium" group, with higher amounts of the following nutrients per 100 g cereal: 100 mg vitamin C, 8,333 IU vitamin A, 18.5 mg vitamin E (α-tocopherol equivalents), and 25.0 mg zinc. If 100 g cereal were consumed per day, the "high" fortification cereal would have provided approximately 50% of the U.S. Daily Value.

Subjects were given two coded boxes for each week of the study, with instructions to consume two to three measured cups (~50-70 g) per day (anytime of the day) for two months. Intake was recorded in daily logs by the subjects/parents, and all unconsumed cereal was returned to investigators for weighing to determine actual intake. To remain in the study, subjects had to consume a minimum of 1,700 g cereal during the study period. Subjects (with parental supervision) were instructed to avoid all other forms of nutrient supplements during the 2-month study.

Diet intake was estimated through 3-day food records pre-study, and then again after one and two months. The study dietitian provided detailed instructions using food models and volume measuring supplies to subjects and parents regarding methods for recording volume and portion size in the 3-day food records, and then entered the information into a computerized dietary analysis system, Food Processor Plus (ESHA Research, Salem, Oregon).

### Blood and saliva sample collection

Children and parents reported to the testing facility between the hours of 7:30-9:30 am having avoided energy intake for the previous 9 h. A 4-min timed saliva sample was first collected followed by a blood sample drawn by trained pediatric phlebotomists. A delayed-type hypersensitivity (DTH) skin test was administered using the Mantoux method with three antigens (*Candida albicans*, mumps antigen, and tetanus toxoid). Each subject reported back to the testing lab two days later for a 48-h measure of skin induration. One month later, subjects returned to the testing lab, and were given a pneumococcal vaccination by medical personnel. At the end of the two-month period, saliva and blood samples were again collected, and the DTH skin test readministered.

### Immune assay measurements

All blood samples were obtained from an antecubital vein from the children while in the supine position after having rested for more than 15 min. Routine complete blood counts were performed by a clinical hematology laboratory (Lab Corp, Burlington, NC), and provided leukocyte subset counts.

#### Lymphocyte subsets

The proportions of T cells (CD3^+^), B cells (CD19^+^), and NK cells (CD3^-^CD16^+^CD56^+^) were determined in whole blood preparations and absolute numbers calculated using CBC data to allow group comparisons on blood concentrations of cells. Lymphocyte phenotyping was accomplished by two-color fluorescent labeling of cell surface antigens with mouse anti-human monoclonal antibodies conjugated to fluoresceinisothiocyanate (FITC) and phycoerythrin (PE) using Simultest monoclonal antibodies and isotype controls (Becton Dickinson, San Jose, CA). For immunophenotyping, 50 μl aliquots of heparinized whole blood from each sample were added to five wells of a 96 well plate. Five μg (diluted in 50 μl RPMI ) of each antibody or isotype control were added to appropriate wells for 20 min on ice in the dark with orbital shaking (170 rpm). The cell suspension was then lysed using 200 μl FACSlyse solution (Becton Dickinson) for 10 min in the dark on ice with shaking. Plates were then centrifuged for five min (Beckman GS-R6 Centrifuge) at 1500 × g. Samples were kept at 4°C in the dark until analyzed by flow cytometry (FacsCalibur, Becton Dickinson, San Jose, CA)

#### Natural killer cell activity (NKCA)

NKCA was assessed using the chromium release assay [34,35]. Peripheral blood mononuclear cells were isolated from heparinized blood by density gradient centrifugation with Ficoll and sodium diatrivoate (American Red Cross, Washington D.C.). ^51^Chromium labeled K562 target cells were then added (1 × 10^4^) to each of the wells containing effector cells to yield 40:1 and 20:1 effector to target (E:T) ratios. The assay was performed in triplicate in "V" bottom microtiter plates (Costar, Cambridge, MA). The microtiter plates were then incubated for 4 h at 37°C in a 5% C02 (in air) incubator. At the end of the incubation, the plates were centrifuged for 5 min at 1500 rpm, the supernatants harvested onto Skatron harvesting frames (Skatron, Sterling, VA), and the level of radioactivity measured in a Packard Tri Carb Liquid Scintillation Analyzer model 2500 TR series (Packard Instruments Company, Meriden, CT). Total release of ^51^Cr was determined by counting an equal aliquot of resuspended cells in 100 μl of 1% triton-X (Sigma, St. Louis, MO). Spontaneous release was determined by counting the radioactivity in the supernatant of labeled target cells cultured in medium alone. The percent lysis was calculated using the mean counts per min (cpm) of triplicate values for each E:T ratio and the following formula:

#### Granulocyte phagocytosis and oxidative burst activity

The phagocytosis assay utilized a FITC-labeled bacteria (*Staphylococcus aureus*; Molecular Probes, Eugene, OR) to quantify the degree of phagocytosis by granulocytes, as described in a previous publication [34]. Briefly, to determine the extent of oxidative burst exhibited by granulocytes, we employed 2',7'-dichlorodihydrofluorescein diacetate (DCF-DA; Molecular Probes), a non-fluorescent molecule which is oxidized to green fluorescent dichlorofluorescein (DCF) as oxygen radicals are generated in the oxidative burst to kill unlabeled *Staphylococcus aureus*. The white blood cell count was acquired using the Becton Dickinson Unopet manual counting protocol. Using two-color flow cytometric immunophenotying (CD45-FITC/CD13,14-PE), the granulocyte percentage was determined. Bioparticle reagents of unlabeled and labeled *Staphylococcus aureus *were suspended into PBS at a working concentration of 3 × 10^5 ^bioparticles/μl. After determining the number of phagocytic cells in 100 μl of whole blood, and adding 15 FITC-labeled bacteria per cell, the mean channel fluorescence (FITC) was analyzed to determine the degree of engulfed bacteria (non-phagocytized bacteria were quenched with ethidium bromide; final concentration of 200 μM). To determine the oxidative burst activities, either DCF-DA (final concentration, 100 μM) (basal activity level), or DCF-DA and unlabeled bacteria (stimulated activity level) were added to 100 μl whole blood. After incubating the samples for 60 min (37°C) in the dark, lysing the RBC, centrifuging, and resuspending the pellets, the samples (10,000 phagocytes) were acquired on the flow cytometer.

#### Salivary IgA

Unstimulated saliva was collected for four min into 5 mL plastic, sterilized vials. Participants were urged to pass as much saliva as possible into the vials during the 4-min timed session. Saliva volume was measured to the nearest 0.1 mL, and then frozen at -80°C until analysis. Salivary IgA was measured by enzyme linked immunosorbent assay (34). The data were expressed as concentration of sIgA (μg ^. ^mL^-1^), concentration of sIgA relative to total protein concentration (μg ^. ^mg^-1^), and salivary immunoglobulin secretion rate (μg ^. ^min^-1^).

#### Delayed-type hypersensitivity (DTH) skin response

The DTH skin response was assessed with use of three antigens, *Candida albicans*, mumps antigen, and tetanus toxoid (diluted 5:1), by the Mantoux method with needle and syringe (Allermed Laboratories, Inc., San Diego, CA; Aventis Pasteur, Swiftwater, PA). The volar surfaces of the left and right arms were cleansed and labeled (mumps antigen and tetanus toxoid on the left arm, Candida albicans on the right). At each site, a needle was inserted into the skin at a 45 degree angle to a depth of <0.2 mm, and 0.1 mL of antigen injected until a 5 mm pea-sized bleb was produced. After 48 h, subjects returned to the lab, and the DTH response at each test site measured. The extent of the induration response was palpated at the reaction area (manifested as firmness and redness), outlined with a black-ink ballpoint pen, and then removed with scotch tape prior to mounting on the skin test record form. The tape impress for each induration was measured across two diameters and averaged.

#### Pneumoccocal vaccination and IgG antibody response

Plasma samples were collected one month before and one month after the children were administered a single 0.5 mL dose of PNEUMOVAX 23 intramuscularly (deltoid muscle) (Merck & Co., Inc., West Point, PA). The plasma samples were assayed for specific IgG antibodies against Pneumococcal Capsular Polysaccharide (PCP) (BINDAZYME™ Anti-PCP IgG Enzyme Immunoassay Kit, MK012, The Binding Site LTD, Birmingham, England). The measuring range of the assay for anti-PCP IgG antibodies levels is 3.3-270 mg/l, with an intra-assay precision of 3.1-5.9% CV and an analytical sensitivity of 0.62 mg/l.

#### Upper respiratory tract infection log

Subjects with parental assistance recorded URTI symptoms on a daily basis in a log using numbered codes. The following health problems were recorded, in accordance with previous investigations by our research team [36]: 1) No health problems; 2) Cold symptoms (runny, stuffy nose, sore throat, coughing, sneezing, colored discharge); 3) Flu symptoms (fever, headache, general aches and pains, fatigue and weakness, chest discomfort, cough); 4) Nausea, vomiting, and/or diarrhea; 5. Muscle, joint, or bone problems/injury; 6) Other health problems. An URTI episode was recorded if cold (#2 item) or flu (#3) symptoms persisted for two days or longer. The primary outcome reported in this study is the total days with URTI symptoms. URTI severity and duration per episode were not monitored or calculated in this study.

### Statistical methods

Data are reported as mean ± SD, and were analyzed using SPSS 11.5 (SPSS Inc., Chicago, IL). The dietary intake data were analyzed using 3 (groups) × 3 (times of measurement) repeated measures ANOVA, with immune data analyzed using a 3 × 2 repeated measures MANOVA. When Box's M suggested a violation of homoscedasticity assumption in MANOVA, Pillais trace statistic was used as the test statistic because it has been shown to be robust against departures from covariance equality. If the group × time interaction P value was ≤ 0.05, a change variable was calculated and compared between groups using Bonferroni adjusted Student's t-tests. URTI data from the daily logs were combined into group averages and the total number of sick days recorded was compared between groups using oneway ANOVA. Chi-square analysis was used to compare the number of children across groups who reported at least one URTI episode during the study period. Power analysis was performed after the study was conducted using the fpower macro in SAS (SAS Institute, Inc., Cary, NC), and revealed that 41 to 142 subjects would be needed (depending on the immune measure) to achieve 80% power. The power analysis is a conservative estimate and was conducted *a posteriori *because data on children for all of the immune measures used in this study were not available prior to the study. We acknowledge that the sample size is considered marginal for this study, but subjects were well randomized, and the results are in accordance with what we and other have observed in adults.

## Results

**Figure 1:**
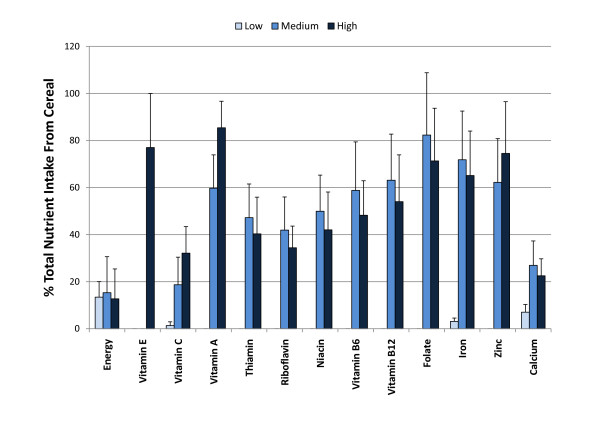
**The percentage contribution of the fortified cereal to total nutrient intake after one and two months supplementation**.

Seventy-three children (55% male, 45% female) started the study, and 65 adhered to all aspects of the study design and were included in the statistical analysis. Groups did not differ significantly in subject characteristics, and data are provided in Table 1 for all 65 subjects completing the study. Subjects consumed an average of 3337 ± 851 g cereal during the two month study, and this quantity did not vary significantly between groups. Nutrient intake (combining self-selected diet and fortified cereal) is summarized in Table 2 for each group. In comparison to the low group, intake of B-complex vitamins, vitamin A, zinc, and iron was elevated in the medium and high groups at 1- and 2-months, with vitamins E and C elevated in the high group. Figure 1 depicts the percentage contribution of the fortified cereal to total nutrient intake. The cereal supplement represented 13.4 ± 6.6, 15.3 ± 4.3, and 12.7 ± 2.9% of total energy intake during the study period for low, medium, and high groups, respectively, and contributed ~20% to 85% of the nutrients listed.

**Table 1:** Subject characteristics (N = 65), mean ± SD*

Variable	Mean ± SD	Range
Age (yrs)	9.8 ± 1.8	7-13
Body mass (kg)	40.7 ± 14.4	23.5-90.3
Stature (m)	1.46 ± 0.11	1.22-1.65
Body mass index (kg/m^2^)	18.9 ± 4.8	13.4-35.9
Sum of 2 skinfolds (mm)	29.5 ± 17.9	9.5-83.8

* For subjects adhering to all aspects of the 2-month research project. Data represent mean values of pre- and post-study measures.

**Table 2:** Nutrient intake data (mean ± SD, from 3-day food records taken pre-study, and after one-month and two-months ingestion of cereal product (low-, medium, and high fortification) in children

Variable	Pre-study	1-month	2-months	Interaction p-value
**Energy (kcal)**				
Low (N = 23)	2313 ± 785	2164 ± 764	2096 ± 851	0.890
Medium (N = 22)	2246 ± 632	2096 ± 535	2084 ± 564	
High (N = 19)	2132 ± 429	2178 ± 577	1952 ± 547	
**Vit E (α-TE, mg)**				
Low	4.30 ± 2.36	4.41 ± 3.72	4.24 ± 2.45	<0.001
Medium	4.20 ± 1.84	3.24 ± 1.97	3.63 ± 1.81	
High	4.28 ± 1.93	13.3 ± 5.08*	13.6 ± 5.11*	
**Vit C (mg)**				
Low	82.3 ± 77.4	68.2 ± 64.6	92.9 ± 89.1	0.002
Medium	79.2 ± 45.8	95.7 ± 86.0	104 ± 95.3	
High	90.2 ± 64.1	178 ± 75.2*	176 ± 58.5	
**Vit A (RE)**				
Low	265 ± 112	239 ± 128	237 ± 144	<0.001
Medium	289 ± 126	572 ± 147*	583 ± 114*	
High	266 ± 117	1727 ± 431*	1657 ± 589*	
**Thiamin (mg)**				
Low	1.62 ± 0.73	1.06 ± 0.58	1.15 ± 0.50	<0.001
Medium	1.57 ± 0.66	1.74 ± 0.55*	1.64 ± 0.47*	
High	1.48 ± 0.35	1.87 ± 0.85*	1.67 ± 0.51*	
**Riboflavin (mg)**				
Low	1.90 ± 0.87	1.47 ± 0.78	1.54 ± 0.72	0.002
Medium	1.90 ± 0.87	2.23 ± 0.80*	2.24 ± 0.77*	
High	1.73 ± 0.63	2.23 ± 0.50*	2.14 ± 0.64*	
**Niacin (mg)**				
Low	19.4 ± 8.4	15.1 ± 9.3	15.4 ± 8.0	0.011
Medium	19.9 ± 9.3	21.5 ± 7.1*	21.4 ± 6.4*	
High	17.9 ± 6.4	23.9 ± 11.8*	21.2 ± 7.8*	
**Vit B6 (mg)**				
Low	1.36 ± 0.57	1.06 ± 0.51	1.16 ± 0.62	<0.001
Medium	1.45 ± 0.72	1.82 ± 0.64*	1.88 ± 0.62*	
High	1.23 ± 0.48	1.98 ± 0.71*	1.81 ± 0.64*	
**Vit B12 (mcg)**				
Low	3.34 ± 1.82	3.30 ± 2.32	2.74 ± 1.43	0.002
Medium	3.80 ± 2.07	5.40 ± 1.79*	5.02 ± 1.70*	
High	3.22 ± 1.63	5.37 ± 1.71*	4.97 ± 1.82*	
**Folate (mcg)**				
Low	288 ± 143	156 ± 79	185 ± 97	<0.001
Medium	265 ± 121	548 ± 174*	499 ± 167*	
High	249 ± 89	513 ± 177*	509 ± 151*	
**Iron (mg)**				
Low	16.2 ± 6.6	10.4 ± 3.8	10.5 ± 3.2	<0.001
Medium	16.1 ± 6.8	26.6 ± 6.6*	24.4 ± 7.2*	
High	14.0 ± 4.2	25.3 ± 7.4*	24.5 ± 6.1*	
**Zinc (mg)**				
Low	9.45 ± 5.72	6.41 ± 3.44	6.75 ± 3.00	<0.001
Medium	9.58 ± 5.87	13.0 ± 4.3*	12.9 ± 3.5*	
High	7.77 ± 3.36	19.0 ± 5.7*	19.9 ± 6.2*	

* = p < 0.05 compared to the low fortification group.

The mean total number of days with URTI during the study period did not differ significantly between groups, and subjects averaged 8.8 ± 10.9, 10.4 ± 6.5, and 7.4 ± 7.2 days with URTI for low, medium, and high groups, respectively (p = 0.556). The percentage of children reporting at least one URTI episode during the study period did not differ between groups: 83%, 90%, and 74% for low, medium, and high groups, respectively (χ^2 ^= 1.78, P = 0.411).

**Figure 2:**
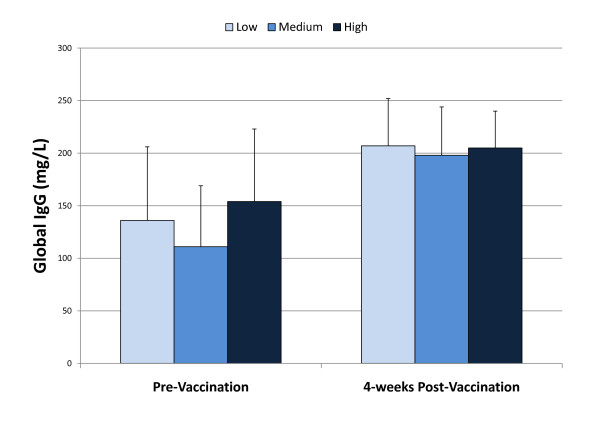
**The 4-week global IgG response to pneumococcal vaccination did not differ between supplementation groups (interaction effect, p = 0.7862)**.

The pattern of change in leukocyte subset counts did not vary significantly between groups (data not shown). Pre- to post-study changes in natural killer cell function (Table 3), granulocyte phagocytosis and oxidative burst activity (Table 4), salivary IgA concentration and secretion rate (Table 5), the global IgG response to pneumococcal vaccination (Figure 2), and DTH response (Table 6) did not differ significantly between groups.

**Table 3:** Natural killer cell counts and activity (NKCA) before and after two-months of cereal ingestion (mean ± SD)*

Variable	Pre-study	Post-study	Treatment interaction p-value
NK lymphocytes (10^9^/L)			0.578
Low	0.39 ± 0.28	0.23 ± 0.13	
Medium	0.49 ± 0.34	0.36 ± 0.20	
High	0.35 ± 0.18	0.29 ± 0.14	
NKCA 40:1 E:T (% lysis)			0.448
Low	44.7 ± 16.8	48.8 ± 16.2	
Medium	47.6 ± 14.1	47.1 ± 16.8	
High	48.4 ± 16.3	48.6 ± 16.3	
NKCA 20:1 E:T (% lysis)			0.349
Low	33.2 ± 16.3	37.0 ± 16.8	
Medium	33.6 ± 12.7	35.5 ± 12.7	
High	38.1 ± 17.1	36.3 ± 13.2	

* Low (N = 24), medium (N = 21), high (N = 18) level of nutrient fortification of the cereal product. Mean ±SD. E:T = effector-to-target ratio.

**Table 4:** Granulocyte phagocytosis and oxidative burst activity before and after two-months of cereal ingestion (mean ± SD)*

Variable Mean flourescence channel	Pre-study	Post-study	Treatment interaction p-value
Oxidative burst activity (DCF^+ ^Granulocytes)			0.184
Low	192 ± 65	136 ± 58	
Medium	184 ± 79	171 ± 119	
High	213 ± 77	141 ± 66	
Phagocytosis (FITC^+ ^Granulocytes)			0.564
Low	459 ± 92	424 ± 84	
Medium	426 ± 109	447 ± 170	
High	442 ± 108	431 ± 93	

* Low (N = 23), medium (N = 20), high (N = 19) = level of nutrient fortification of the cereal product. DCF = dichlorofluorescein; FITC = fluorescein isothiocyanate.

**Table 5:** Salivary IgA concentration and secretion rate before and after two-months of cereal ingestion (mean ±SD)*

Variable	Pre-study	Post-study	Treatment interaction p-value
Saliva IgA concentration (μg ^. ^mL^-1^)			0.461
Low	333 ± 181	314 ± 152	
Medium	292 ± 143	302 ± 151	
High	315 ± 159	289 ± 158	
Saliva protein IgA concentration (μg ^. ^mg^-1^)			0.675
Low	471 ± 162	484 ± 156	
Medium	444 ± 170	419 ± 155	
High	535 ± 224	499 ± 187	
Saliva IgA secretion rate (μg ^. ^min^-1^)			0.476
Low	173 ± 135	220 ± 163	
Medium	185 ± 118	233 ± 129	
High	207 ± 118	212 ± 74	

* Low (N = 21), medium (N = 24), high (N = 19) = level of nutrient fortification of the cereal product.

**Table 6:** Delayed-type hypersensitivity (DTH) skin response before and after two-months of cereal ingestion (mean ±SD)*

Variable	Pre-study	Post-study	Treatment interaction p-value
DTH skin response (mm)			0.812
Low	23.5 ± 10.1	12.8 ± 7.5	
Medium	22.5 ± 11.3	16.4 ± 12.4	
High	24.3 ± 13.7	12.6 ± 6.3	

* DTH skin response represents the sum of three skin tests: tetanus, mumps, and candin. Low (N = 21), medium (N = 21), high (N = 19) = level of nutrient fortification of the cereal product.

## Discussion

Despite significant improvements in dietary intake of immune-related nutrients through ingestion of a fortified cereal (~60 g/day) during a 2-month winter period, URTI rates and pre- to- post-study changes in all immune function measures did not differ between groups. These data do not support the use of micronutrient fortified breakfast cereals by healthy, growing children for the purpose of augmenting innate or adaptive immune function and lowering URTI risk.

Although dietary nutrients are important for immunocompetence in all humans [1], related benefits from micronutrient supplements are most often reported in populations with immune dysreglation such as malnourished children, the frail elderly, diseased individuals, and surgical or trauma patients [5-13]. Among healthy adults, immune function remains robust over a wide range of diet quality and nutrient intake, and adding supplemental nutrients beyond what is obtained in the traditional food supply has little if any discernable influence on immunity and infection rates [14-22]. In a double-blinded, randomized, placebo-controlled study of 138 healthy adults aged 40-80 years, Wolvers et al. [21] reported no effect of 10-weeks supplementation with a micronutrient mix (vitamins E and C, β-carotene, and zinc) on a range of immunological measures including the antibody response to vaccination, phagocytosis and oxidative burst activity, and lymphocyte proliferation. An enhanced DTH response was reported, but this benefit was limited to the older subjects, similar to data previously reported by Bogden et al. [37]. Other studies of healthy adults with uncompromised immune systems using single-nutrient supplements such as zinc, selenium, or vitamin E have also reported negligible immune benefits, although tocotrienol supplementation may improve the immune response to a vaccine challenge [19-24,38].

To our knowledge, our study is the first to investigate the influence of micronutrient supplementation through breakfast cereal fortification on immune function and URTI in healthy, school-aged youth. Kutukculer et al. [32] studied the effect of acute, large dose vitamin A and E supplementation on the IgG response to tetanus toxoid immunization in healthy infants, and reported no supplementation effect on the antibody response. Several other studies have reported positive immunological responses and reductions in acute lower respiratory infection and diarrhea to nutrient supplementation in malnourished or infected young children [25-31]. Thus the limited data available on the relationship between micronutrient intake and supplementation on immune function in children is consistent with data collected in adult populations: supplemental micronutrients are efficacious among those suffering from syndromes related to immune dysfunction but unlikely to augment immune function or decrease infection risk among well nourished and healthy individuals.

Among our "medium" and "high" supplement groups, ingestion of the fortified breakfast cereal represented ~20-85% of total dietary intake for zinc, iron, vitamin B6, folate (and other B complex vitamins), and antioxidant vitamins. Supplemental capsules containing a micronutrient mixture would have provided a greater quantity, but this study was designed to test the utility and efficacy of using a fortified breakfast cereal delivery system. The volume of breakfast cereal consumed per day by our young subjects (mean of ~60 g or 2.7 cups) over a two month period approached the maximum amount they could tolerate. For the five key immune-related nutrients in the "high" fortification group--vitamins A, C, and E, zinc, iron--supplementation with the fortified cereal product by the children during the study provided approximately 30% of the U.S. Daily Value above typical food intake. Thus at the nutrient density used in this study, the strategy of using fortified breakfast cereal to improve immune function and lower URTI risk in healthy children is not recommended.

## Conclusions

In conclusion, our hypothesis of URTI incidence reduction and immune modulation in healthy, school-aged children through ingestion of a breakfast cereal fortified with immune-related nutrients was not supported. We measured URTI rates and conducted a variety of *in vitro *and *in vivo *measures of both innate and adaptive immune function, and failed to find any significant group differences during a 2-month winter period. Thus within the context of this investigation, our data do not support the use of immune benefit claims for healthy, school-aged children from ingestion of fortified breakfast cereal.

## Competing interests

The authors declare that they have no competing interests.

## Authors' contributions

NDC was the primary investigator for this study, managed all aspects of subject recruitment and scheduling, data collection, sample analysis, and manuscript preparation. HDA (immunologist) coordinated all sample collection and immune assays, and participated in preparation of the manuscript. SW assisted in the statistical analysis. All authors have read and approved the final manuscript

## References

[B1] Maggini S Wintergerst ES Beveridge S Hornig DH Selected vitamins and trace elements support immune function by strengthening epithelial barriers and cellular and humoral immune responses Br J Nutr 2007 98 Suppl 1 S29 35 17922955 10.1017/S0007114507832971

[B2] Prasad AS Beck FW Bao B Fitzgerald JT Snell DC Steinberg JD Cardozo LJ Zinc supplementation decreases incidence of infections in the elderly: effect of zinc on generation of cytokines and oxidative stress Am J Clin Nutr 2007 85 837 844 17344507 10.1093/ajcn/85.3.837

[B3] Wichers H Immunomodulation by food: promising concept for mitigating allergic disease? Anal Bioanal Chem 2009 395 37 45 10.1007/s00216-009-2838-1 19455311 PMC2724636

[B4] Keusch GT The history of nutrition: malnutrition, infection and immunity J Nutr 2003 133 336S 340S 12514322 10.1093/jn/133.1.336S

[B5] Cunningham-Rundles S McNeeley DF Moon A Mechanisms of nutrient modulation of the immune response J Allergy Clin Immunol 2005 115 1119 1128 10.1016/j.jaci.2005.04.036 15940121

[B6] Girodon F Galan P Mong Boutron-Ruault MC Brunet-Lecomte P Preziosi P Arnaud J Manuguerra JC Herchberg S Impact of trace elements and vitamin supplementation on immunity and infections in institutionalized elderly patients: a randomized controlled trial. MIN. VIT. AOX. geriatric network Arch Intern Med 1999 159 748 754 10.1001/archinte.159.7.748 10218756

[B7] Berger MM Binnert C Chiolero RL Taylor W Raffoul W Cayeux MC Benathan M Shenkin A Tappy L Trace element supplementation after major burns increases burned skin trace element concentrations and modulates local protein metabolism but not whole-body substrate metabolism Am J Clin Nutr 2007 85 1301 1306 17490966 10.1093/ajcn/85.5.1301

[B8] Meydani SN Meydani M Blumberg JB Leka LS Siber G Loszewski R Thompson C Pedrosa MC Diamond RD Stollar BD Vitamin E supplementation and in vivo immune response in healthy elderly subjects. A randomized controlled trial JAMA 1997 277 1380 1386 10.1001/jama.277.17.1380 9134944

[B9] Calder PC Immunonutrition in surgical and critically ill patients Br J Nutr 2007 98 Suppl 1 S133 S1339 17922951 10.1017/S0007114507832909

[B10] Deloria-Knoll M Steinhoff M Semba RD Nelson K Vlahov D Meinert CL Effect of zinc and vitamin A supplementation on antibody responses to a pneumococcal conjugate vaccine in HIV-positive injection drug users: a randomized trial Vaccine 2006 24 1670 1679 10.1016/j.vaccine.2005.09.047 16256250

[B11] Hamer DH Sempértegui F Estrella B Tucker KL Rodríguez A Egas J Dallal GE Selhub J Griffiths JK Meydani SN Micronutrient deficiencies are associated with impaired immune response and higher burden of respiratory infections in elderly Ecuadorians J Nutr 2009 139 113 119 19056665 10.3945/jn.108.095091 PMC2646211

[B12] Ravaglia G Forti P Maioli F Bastagli L Facchini A Mariani E Savarino L Sassi S Cucinotta D Lenaz G Effect of micronutrient status on natural killer cell immune function in healthy free-living subjects aged ≥90 y Am J Clin Nutr 2000 71 590 598 10648276 10.1093/ajcn/71.2.590

[B13] Asprer JM Llido LO Sinamban R Schlotzer E Kulkarni H Effect on immune indices of preoperative intravenous glutamine dipeptide supplementation in malnourished abdominal surgery patients in the preoperative and postoperative periods Nutrition 2009 25 920 925 10.1016/j.nut.2009.01.014 19647623

[B14] Payette H Rola-Pleszczynski M Ghadirian P Nutrition factors in relation to cellular and regulatory immune variables in a free-living elderly population Am J Clin Nutr 1990 52 927 932 2239770 10.1093/ajcn/52.5.927

[B15] Goodwin JS Garry PJ Lack of correlation between indices of nutritional status and immunologic function in elderly humans J Gerontol 1988 43 M46 M49 3346522 10.1093/geronj/43.2.m46

[B16] Boynton A Neuhouser ML Wener MH Wood B Sorensen B Chen-Levy Z Kirk EA Yasui Y Lacroix K McTiernan A Ulrich CM Associations between healthy eating patterns and immune function or inflammation in overweight or obese postmenopausal women Am J Clin Nutr 2007 86 1445 1455 17991658 10.1093/ajcn/86.5.1445

[B17] Neuman MI Willett WC Curhan GC Vitamin and micronutrient intake and the risk of community-acquired pneumonia in US women Am J Med 2007 120 330 336 10.1016/j.amjmed.2006.06.045 17398227 PMC1964883

[B18] Merchant AT Curhan G Bendich A Singh VN Willett WC Fawzi WW Vitamin intake is not associated with community-acquired pneumonia in U.S. men J Nutr 2004 134 439 444 14747686 10.1093/jn/134.2.439

[B19] Hawkes WC Hwang A Alkan Z The effect of selenium supplementation on DTH skin responses in healthy North American men J Trace Elem Med Biol 2009 23 272 280 10.1016/j.jtemb.2009.04.002 19747623

[B20] Radhakrishnan AK Lee AL Wong PF Kaur J Aung H Nesaretnam K Daily supplementation of tocotrienol-rich fraction or alpha-tocopherol did not induce immunomodulatory changes in healthy human volunteers Br J Nutr 2009 101 810 815 10.1017/S0007114508039998 18702848

[B21] Wolvers DA van Herpen-Broekmans WM Logman MH van der Wielen RP Albers R Effect of a mixture of micronutrients, but not of bovine colostrum concentrate, on immune function parameters in healthy volunteers: a randomized placebo-controlled study Nutr J 2006 5 28 10.1186/1475-2891-5-28 17118191 PMC1676011

[B22] Boardley D Fahlman M Micronutrient supplementation does not attenuate seasonal decline of immune system indexes in well-nourished elderly women: a placebo-controlled study J Am Diet Assoc 2000 100 356 359 10.1016/S0002-8223(00)00108-5 10719412

[B23] Hodkinson CF Kelly M Alexander HD Bradbury I Robson PJ Bonham MP O'Connor JM Coudray C Strain JJ Wallace JM Effect of zinc supplementation on the immune status of healthy older individuals aged 55-70 years: the ZENITH Study J Gerontol A Biol Sci Med Sci 2007 62 598 608 17595415 10.1093/gerona/62.6.598

[B24] Veverka DV Wilson C Martinez MA Wenger R Tamosuinas A Use of zinc supplements to reduce upper respiratory infections in United States Air Force Academy cadets Complement Ther Clin Pract 2009 15 91 95 10.1016/j.ctcp.2009.02.006 19341987

[B25] Sazawal S Black RE Jalla S Mazumdar S Sinha A Bhan MK Zinc supplementation reduces the incidence of acute lower respiratory infections in infants and preschool children: a double-blind, controlled trial Pediatrics 1998 102 1 Pt 1 1 5 9651405 10.1542/peds.102.1.1

[B26] Roth DE Caulfield LE Ezzati M Black RE Acute lower respiratory infections in childhood: opportunities for reducing the global burden through nutritional interventions Bull World Health Organ 2008 86 356 364 10.2471/BLT.07.049114 18545738 PMC2647440

[B27] Sempértegui F Estrella B Camaniero V Betancourt V Izurieta R Ortiz W Fiallo E Troya S Rodríguez A Griffiths JK The beneficial effects of weekly low-dose vitamin A supplementation on acute lower respiratory infections and diarrhea in Ecuadorian children Pediatrics 1999 104 1 e1 10.1542/peds.104.1.e1 10390287

[B28] Raqib R Roy SK Rahman MJ Azim T Ameer SS Chisti J Andersson J Effect of zinc supplementation on immune and inflammatory responses in pediatric patients with shigellosis Am J Clin Nutr 2004 79 444 450 14985220 10.1093/ajcn/79.3.444

[B29] Ahmed J Zaman MM Ali SM Immunological response to antioxidant vitamin supplementation in rural Bangladeshi school children with group A streptococcal infection Asia Pac J Clin Nutr 2004 13 226 230 15331332

[B30] Nchito M Geissler PW Mubila L Friis H Olsen A The effect of iron and multi-micronutrient supplementation on Ascaris lumbricoides reinfection among Zambian schoolchildren Trans R Soc Trop Med Hyg 2009 103 229 236 10.1016/j.trstmh.2008.08.005 18937957

[B31] Lin J Song F Yao P Yang X Li N Sun S Lei L Liu L Effect of vitamin A supplementation on immune function of well-nourished children suffering from vitamin A deficiency in China Eur J Clin Nutr 2008 62 1412 1418 10.1038/sj.ejcn.1602881 17684522

[B32] Kutukculer N Akil T Egemen A Kurugöl Z Akşit S Ozmen D Turgan N Bayindir O Cağlayan S Adequate immune response to tetanus toxoid and failure of vitamin A and E supplementation to enhance antibody response in healthy children Vaccine 2000 18 2979 2984 10.1016/S0264-410X(00)00097-9 10825599

[B33] Lohman TG Applicability of body composition techniques and constants for children and youth Ex Sport Sci Rev 1986 14 325 357 3525188

[B34] Nieman DC Henson DA Nehlsen-Cannarella SL Utter A Butterworth DE Fagoaga OR Influence of obesity on immune function J Am Diet Assoc 1999 99 294 299 10.1016/S0002-8223(99)00077-2 10076580

[B35] Whiteside TL Bryant J Day R Herberman RB Natural killer cytotoxicity in the diagnosis of immune dysfunction: Criteria for a reproducible assay J Clin Lab Anal 1990 4 102 114 10.1002/jcla.1860040207 2179501

[B36] Nieman DC Nehlsen-Cannarella SL Henson DA Koch AJ Butterworth DE Fagoaga OR Utter A Immune response to exercise training and/or energy restriction in obese females Med Sci Sports Exerc 1998 30 679 686 10.1097/00005768-199805000-00006 9588608

[B37] Bogden JD Bendich A Kemp FW Bruening KS Shurnick JH Denny T Baker H Louria DB Daily micronutrient supplements enhance delayed-hypersensitivity skin test responses in older people Am J Clin Nutr 1994 60 437 447 8074079 10.1093/ajcn/60.3.437

[B38] Mahalingam D Radhakrishnan AK Amom Z Ibrahim N Nesaretnam K Effects of supplementation with tocotrienol-rich fraction on immune response to tetanus toxoid immunization in normal healthy volunteers Eur J Clin Nutr 2011 65 63 69 10.1038/ejcn.2010.184 20859299

